# (2-{[1-(Pyridin-2-yl)ethyl­idene]amino­meth­yl}pyridine-κ^3^ *N*,*N*′,*N*′′)bis­(thio­cyanato-κ*N*)zinc

**DOI:** 10.1107/S1600536811043984

**Published:** 2011-10-29

**Authors:** Chen-Yi Wang, Jing-Fen Li, Xiang Wu, Hai-Yu Tu, Pei-Fei Zhu

**Affiliations:** a Department of Chemistry, Huzhou University, Huzhou 313000, People’s Republic of China

## Abstract

The complete mol­ecule of the title mononuclear zinc(II) complex, [Zn(NCS)_2_(C_13_H_13_N_3_)], is generated by crystallographic twofold symmetry, with the metal atom lying on the rotation axis. The pendant methyl group of the ligand is statistically disordered over two sites. The Zn^2+^ cation is coordinated by the *N*,*N*′,*N*′′-tridentate Schiff base ligand, and by two thio­cyanate N atoms, forming a distorted ZnN_5_ trigonal–bipyramidal geometry.

## Related literature

For Schiff-base complexes reported by us, see: Wang & Ye (2011 ▶); Wang (2009 ▶); Wang *et al.* (2011 ▶). For similar zinc(II) complexes, see: Wang (2010 ▶); Huang (2011 ▶); Ikmal Hisham *et al.* (2011 ▶); Wang (2011 ▶).
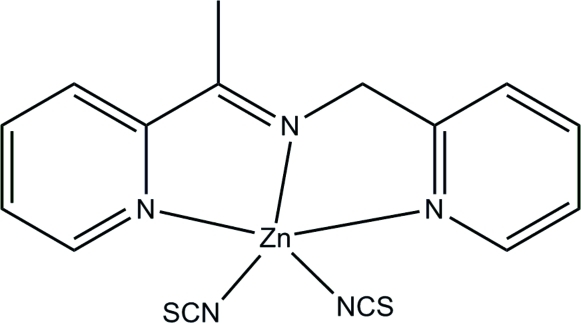


## Experimental

### 

#### Crystal data

[Zn(NCS)_2_(C_13_H_13_N_3_)]
*M* *_r_* = 392.79
Monoclinic, 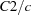

*a* = 14.272 (3) Å
*b* = 8.633 (3) Å
*c* = 15.338 (3) Å
β = 110.945 (2)°
*V* = 1764.9 (8) Å^3^
*Z* = 4
Mo *K*α radiation
μ = 1.63 mm^−1^
*T* = 298 K
0.17 × 0.13 × 0.12 mm

#### Data collection

Bruker SMART CCD diffractometer
Absorption correction: multi-scan (*SADABS*; Sheldrick, 1996 ▶) *T*_min_ = 0.769, *T*_max_ = 0.828
3113 measured reflections
1838 independent reflections
1395 reflections with *I* > 2σ(*I*)
*R*_int_ = 0.023

#### Refinement

*R*[*F* ^2^ > 2σ(*F* ^2^)] = 0.040
*wR*(*F* ^2^) = 0.109
*S* = 1.07
1838 reflections
111 parameters
H-atom parameters constrained
Δρ_max_ = 0.59 e Å^−3^
Δρ_min_ = −0.40 e Å^−3^

### 

Data collection: *SMART* (Bruker, 1998 ▶); cell refinement: *SAINT* (Bruker, 1998 ▶); data reduction: *SAINT*; program(s) used to solve structure: *SHELXS97* (Sheldrick, 2008 ▶); program(s) used to refine structure: *SHELXL97* (Sheldrick, 2008 ▶); molecular graphics: *SHELXTL* (Sheldrick, 2008 ▶); software used to prepare material for publication: *SHELXTL*.

## Supplementary Material

Crystal structure: contains datablock(s) global, I. DOI: 10.1107/S1600536811043984/hb6391sup1.cif

Structure factors: contains datablock(s) I. DOI: 10.1107/S1600536811043984/hb6391Isup2.hkl

Additional supplementary materials:  crystallographic information; 3D view; checkCIF report

## Figures and Tables

**Table:** 

Zn1—N3	1.970 (3)
Zn1—N2	2.076 (4)
Zn1—N1	2.156 (3)

**Table:** 

N1^i^—Zn1—N1	152.16 (16)

Symmetry code: (i) 
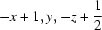
.

**Fig. 1.**
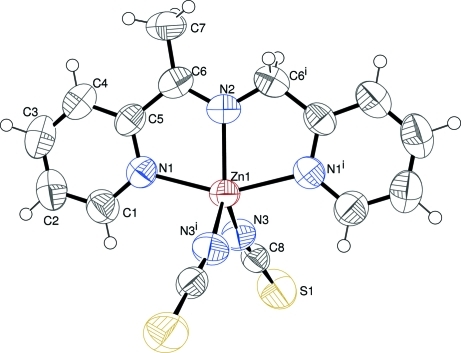
The molecular structure of the title compound, showing displacement ellipsoids drawn at the 50% probability level. Symmetry code: (i) 1–x, y, 1/2–z.

## supplementary crystallographic information

### Comment

Schiff bases and their complexes have been widely studied for their synthesis, structures, and biological activities. As part of our investigations into Schiff base complexes (Wang & Ye, 2011; Wang, 2009; Wang *et al.*, 2011), we have synthesized the title compound, a new mononuclear zinc(II) complex, Fig. 1. The Zn atom in the complex is five-coordinated by the three N atoms of the Schiff base ligand, and by two thiocyanate N atoms, forming a distorted square pyramidal geometry. The Zn–N bond lengths (Table 1) are typical and are comparable with those observed in other similar zinc(II) complexes (Wang, 2010; Huang, 2011; Ikmal Hisham *et al.*, 2011; Wang, 2011).

### Experimental

2-Acetylpyridine (1.0 mmol, 0.121 g) and 2-aminomethylpyridine (1.0 mmol, 0.108 g) were dissolved in MeOH (30 ml), to the mixture was added with stirring an aqueous solution (5 ml) of ammonium thiocyanate (2.0 mmol, 0.152 g) and zinc acetate dihydrate (1.0 mmol, 0.220 g). The final mixture was stirred at room temperature for 10 min to give a clear colorless solution. After keeping the solution in air for a week, colorless block-shaped crystals were formed at the bottom of the vessel.

### Refinement

H atoms were placed in geometrically idealized positions and constrained to ride on their parent atoms, with C—H distances in the range 0.93–0.96 Å, and with *U*_iso_(H) set at 1.2*U*_eq_(C) and 1.5*U*_eq_(C8). The methyl group is disordered over a twofold rotation axis symmetry.

### Crystal data

**Table:**

[Zn(NCS)_2_(C_13_H_13_N_3_)]	*F*(000) = 800
*M**_r_* = 392.79	*D*_x_ = 1.478 Mg m^−^^3^
Monoclinic, *C*2/*c*	Mo *K*α radiation, λ = 0.71073 Å
*a* = 14.272 (3) Å	Cell parameters from 1062 reflections
*b* = 8.633 (3) Å	θ = 2.6–24.5°
*c* = 15.338 (3) Å	µ = 1.63 mm^−^^1^
β = 110.945 (2)°	*T* = 298 K
*V* = 1764.9 (8) Å^3^	Block, colorless
*Z* = 4	0.17 × 0.13 × 0.12 mm

### Data collection

**Table:**

Bruker SMART CCD diffractometer	1838 independent reflections
Radiation source: fine-focus sealed tube	1395 reflections with *I* > 2σ(*I*)
graphite	*R*_int_ = 0.023
ω scan	θ_max_ = 27.0°, θ_min_ = 2.8°
Absorption correction: multi-scan (*SADABS*; Sheldrick, 1996)	*h* = −17→13
*T*_min_ = 0.769, *T*_max_ = 0.828	*k* = −10→7
3113 measured reflections	*l* = −19→18

### Refinement

**Table:**

Refinement on *F*^2^	Primary atom site location: structure-invariant direct methods
Least-squares matrix: full	Secondary atom site location: difference Fourier map
*R*[*F*^2^ > 2σ(*F*^2^)] = 0.040	Hydrogen site location: inferred from neighbouring sites
*wR*(*F*^2^) = 0.109	H-atom parameters constrained
*S* = 1.07	*w* = 1/[σ^2^(*F*_o_^2^) + (0.0552*P*)^2^ + 0.5867*P*] where *P* = (*F*_o_^2^ + 2*F*_c_^2^)/3
1838 reflections	(Δ/σ)_max_ < 0.001
111 parameters	Δρ_max_ = 0.59 e Å^−^^3^
0 restraints	Δρ_min_ = −0.40 e Å^−^^3^

### Special details

**Table:**

Geometry. All e.s.d.'s (except the e.s.d. in the dihedral angle between two l.s. planes) are estimated using the full covariance matrix. The cell e.s.d.'s are taken into account individually in the estimation of e.s.d.'s in distances, angles and torsion angles; correlations between e.s.d.'s in cell parameters are only used when they are defined by crystal symmetry. An approximate (isotropic) treatment of cell e.s.d.'s is used for estimating e.s.d.'s involving l.s. planes.
Refinement. Refinement of *F*^2^ against ALL reflections. The weighted *R*-factor *wR* and goodness of fit *S* are based on *F*^2^, conventional *R*-factors *R* are based on *F*, with *F* set to zero for negative *F*^2^. The threshold expression of *F*^2^ > σ(*F*^2^) is used only for calculating *R*-factors(gt) *etc*. and is not relevant to the choice of reflections for refinement. *R*-factors based on *F*^2^ are statistically about twice as large as those based on *F*, and *R*- factors based on ALL data will be even larger.

### Fractional atomic coordinates and isotropic or equivalent isotropic displacement parameters (Å^2^)

**Table:**

	*x*	*y*	*z*	*U*_iso_*/*U*_eq_	Occ. (<1)
Zn1	0.5000	0.64025 (5)	0.2500	0.0552 (2)	
S1	0.28816 (12)	0.97184 (15)	0.33736 (10)	0.1135 (5)	
N1	0.42235 (19)	0.5802 (3)	0.10557 (18)	0.0607 (6)	
N2	0.5000	0.3998 (4)	0.2500	0.0635 (10)	
N3	0.3999 (2)	0.7781 (3)	0.2707 (2)	0.0700 (7)	
C1	0.3860 (3)	0.6806 (4)	0.0353 (3)	0.0768 (10)	
H1	0.3924	0.7862	0.0481	0.092*	
C2	0.3395 (3)	0.6326 (5)	−0.0556 (3)	0.0885 (12)	
H2	0.3137	0.7045	−0.1034	0.106*	
C3	0.3318 (3)	0.4777 (6)	−0.0745 (3)	0.0943 (13)	
H3	0.3019	0.4425	−0.1355	0.113*	
C4	0.3682 (3)	0.3755 (5)	−0.0031 (3)	0.0822 (11)	
H4	0.3624	0.2695	−0.0147	0.099*	
C5	0.4139 (2)	0.4294 (4)	0.0865 (2)	0.0622 (8)	
C6	0.4563 (3)	0.3228 (4)	0.1674 (3)	0.0693 (9)	0.50
C7	0.4455 (7)	0.1605 (7)	0.1517 (7)	0.093 (3)	0.50
H7A	0.5040	0.1085	0.1931	0.140*	0.50
H7B	0.4382	0.1386	0.0882	0.140*	0.50
H7C	0.3873	0.1248	0.1631	0.140*	0.50
C6'	0.4563 (3)	0.3228 (4)	0.1674 (3)	0.0693 (9)	0.50
H6'1	0.4030	0.2573	0.1720	0.083*	0.50
H6'2	0.5059	0.2564	0.1566	0.083*	0.50
C8	0.3551 (3)	0.8592 (4)	0.2993 (2)	0.0608 (8)	

### Atomic displacement parameters (Å^2^)

**Table:**

	*U*^11^	*U*^22^	*U*^33^	*U*^12^	*U*^13^	*U*^23^
Zn1	0.0596 (3)	0.0491 (3)	0.0609 (3)	0.000	0.0265 (2)	0.000
S1	0.1452 (12)	0.1040 (9)	0.1163 (9)	0.0557 (8)	0.0772 (9)	0.0132 (7)
N1	0.0577 (16)	0.0650 (15)	0.0630 (16)	0.0002 (13)	0.0260 (12)	−0.0040 (12)
N2	0.064 (2)	0.054 (2)	0.074 (3)	0.000	0.0264 (19)	0.000
N3	0.0687 (18)	0.0654 (17)	0.0806 (19)	0.0099 (14)	0.0323 (15)	−0.0043 (15)
C1	0.083 (3)	0.076 (2)	0.073 (2)	0.0081 (19)	0.029 (2)	0.0052 (18)
C2	0.088 (3)	0.112 (3)	0.064 (2)	0.011 (2)	0.024 (2)	0.007 (2)
C3	0.092 (3)	0.123 (4)	0.064 (2)	0.008 (3)	0.023 (2)	−0.018 (2)
C4	0.084 (3)	0.084 (3)	0.076 (2)	−0.003 (2)	0.026 (2)	−0.021 (2)
C5	0.0584 (19)	0.0672 (19)	0.065 (2)	−0.0004 (16)	0.0263 (16)	−0.0130 (16)
C6	0.068 (2)	0.063 (2)	0.079 (2)	−0.0014 (16)	0.0287 (18)	−0.0115 (16)
C7	0.115 (7)	0.052 (4)	0.104 (6)	0.001 (4)	0.027 (5)	−0.003 (4)
C6'	0.068 (2)	0.063 (2)	0.079 (2)	−0.0014 (16)	0.0287 (18)	−0.0115 (16)
C8	0.064 (2)	0.0590 (18)	0.0593 (18)	0.0067 (16)	0.0224 (15)	0.0074 (15)

### Geometric parameters (Å, °)

**Table:**

Zn1—N3^i^	1.970 (3)	C1—H1	0.9300
Zn1—N3	1.970 (3)	C2—C3	1.364 (5)
Zn1—N2	2.076 (4)	C2—H2	0.9300
Zn1—N1^i^	2.156 (3)	C3—C4	1.356 (6)
Zn1—N1	2.156 (3)	C3—H3	0.9300
S1—C8	1.612 (3)	C4—C5	1.374 (5)
N1—C5	1.330 (4)	C4—H4	0.9300
N1—C1	1.336 (4)	C5—C6	1.489 (5)
N2—C6'^i^	1.368 (4)	C6—C7	1.421 (7)
N2—C6^i^	1.368 (4)	C7—H7A	0.9600
N2—C6	1.368 (4)	C7—H7B	0.9600
N3—C8	1.136 (4)	C7—H7C	0.9600
C1—C2	1.376 (5)		
			
N3^i^—Zn1—N3	105.66 (17)	N1—C1—C2	122.0 (4)
N3^i^—Zn1—N2	127.17 (9)	N1—C1—H1	119.0
N3—Zn1—N2	127.17 (9)	C2—C1—H1	119.0
N3^i^—Zn1—N1^i^	100.08 (11)	C3—C2—C1	118.9 (4)
N3—Zn1—N1^i^	96.64 (11)	C3—C2—H2	120.5
N2—Zn1—N1^i^	76.08 (8)	C1—C2—H2	120.5
N3^i^—Zn1—N1	96.64 (11)	C4—C3—C2	119.2 (4)
N3—Zn1—N1	100.08 (11)	C4—C3—H3	120.4
N2—Zn1—N1	76.08 (8)	C2—C3—H3	120.4
N1^i^—Zn1—N1	152.16 (16)	C3—C4—C5	119.6 (4)
C5—N1—C1	118.6 (3)	C3—C4—H4	120.2
C5—N1—Zn1	115.8 (2)	C5—C4—H4	120.2
C1—N1—Zn1	125.6 (3)	N1—C5—C4	121.7 (3)
C6'^i^—N2—C6^i^	0.0 (3)	N1—C5—C6	116.3 (3)
C6'^i^—N2—C6	121.8 (4)	C4—C5—C6	122.0 (3)
C6^i^—N2—C6	121.8 (4)	N2—C6—C7	128.5 (5)
C6'^i^—N2—Zn1	119.1 (2)	N2—C6—C5	112.8 (3)
C6^i^—N2—Zn1	119.1 (2)	C7—C6—C5	118.7 (5)
C6—N2—Zn1	119.1 (2)	N3—C8—S1	178.2 (3)
C8—N3—Zn1	167.1 (3)		

Symmetry codes: (i) −*x*+1, *y*, −*z*+1/2.
